
JOURNAL INFORMATION
==============================
NLM Title Abbreviation: ScientificWorldJournal
Journal ID: ScientificWorldJournal
NLM Journal ID: 101131163
Journal ID: TSWJ

The Scientific World Journal

ISSN: 2356-6140
EISSN: 1537-744X
Publisher: Wiley

ARTICLE INFORMATION
==============================
PMCID: PMC6084113
DOI: 10.1100/tsw.2001.439
Article ID: 708934
Article version: 1
Subjects: Extended Abstract

Altered Neuroadaptation in Opiate Dependence and Neurogenic Inflammatory Nociception in αCGRP Deficient Mice

Salmon A.-M.
Damaj I. M.
Marubio L. M.
Epping-Jordan M. P.
Pich E. Merlo
Changeux J.-P.
Institut Pasteur, Neurobiologie Moleculaire-Biotechnologie, 25 Rue du Dr. Roux, 75015 Paris, France
Publication date: 2001
Electronic publication date: 2001 Oct 17
Volume: 1
First page: 22
Last page: 22
Copyright: Copyright © 2001 A.-M. Salmon et al.
Copyright year: 2001
License: This is an open access article distributed under the Creative Commons Attribution License, which permits unrestricted use, distribution, and reproduction in any medium, provided the original work is properly cited.
License URL: https://creativecommons.org/licenses/by/3.0/

==============================
4th International Meeting on Calcitonin Gene-Related Peptide (CGRP)
*TheScientificWorld* (2001) 1(S1), 22
ISSN 1532-2246; DOI 10.1100/tsw.2001.439

### ALTERED NEUROADAPTATION IN OPIATE DEPENDENCE AND NEUROGENIC INFLAMMATORY NOCICEPTION IN αCGRP DEFICIENT MICE

A.-M. Salmon, I.M. Damaj, L.M.Marubio, M.P. Epping-Jordan, E. Merlo Pich, and J.-P. Changeux
Institut Pasteur, Neurobiologie Moleculaire-Biotechnologie, 25 Rue du Dr. Roux, 75015 Paris, France

αCGRP is expressed in a variety of cell types in both central and peripheral nervous system[1]. Among its various functions, it is involved in the complex process of pain signaling[2,3]. Yet, the precise contribution of αCGRP is still unclear.

Using αCGRP deficient mice (-/-), we show that the lack of αCGRP underlies an attenuated response to chemical pain and inflammation[4]. In other words, αCGRP is critical for the production and, possibly, the transmission of pain signals associated with neurogenic inflammation. Furthermore, αCGRP -/- mice present a reduction in the antinociceptive response to morphine, indicating a modulatory role (agonist) of αCGRP in opioid pathways[5]. In contrast, the antinociceptive response to nicotine is potentiated, showing a negative modulation of nAChR function by this neuropeptide[4]. Thus, αCGRP has a complex role in the modulation of analgesic drug pathways.

Moreover, αCGRP -/- mice do not show changes in morphine self-administration and tolerance, but display a marked decrease in morphine withdrawal signs[4]. We suggest that αCGRP may influence the contribution of peripheral signals to the aversive emotional state occurring in the opiate dependence syndrome.

Taken together, these results show that αCGRP plays a critical role in mediating both chemical inflammatory pain and sensitivity to morphine withdrawal. Antagonists of αCGRP may thus offer novel therapeutics for the treatment of pain and drug addiction.

### REFERENCES

1. Rosenfeld, M.G., Amara, S.G., and Evans, R.M. (1984) Science 225, 1315–1320.
2. Hughes, S.R. and Brain, S.D. (1991) J. Pharmacol. 104, 738–742.
3. Gibbins, I.L., Furness, J.B., and Costa, M. (1987) Cell Tissue Res. 248, 417–437.
4. Salmon, A.M. et al. (2001) Nature Neurosci. 4, 357–358.
5. Salmon, A.M. et al. (1999) NeuroReport 10, 849–854.
